# A Set of Proximal Regulatory Elements Contribute to the Transcriptional Activity of the Human Lipoprotein Lipase Promoter

**DOI:** 10.3390/cimb46110788

**Published:** 2024-11-18

**Authors:** Nasmah K. Bastaki, Taybha A. Albarjes, Afnan K. Mohamed, Noorhan H. Sabri, Suzanne A. Al-Bustan

**Affiliations:** Department of Biological Science, Faculty of Science, Kuwait University, Kuwait City 13060, Kuwait

**Keywords:** lipoprotein lipase, human, promotor, luciferase, regulatory elements

## Abstract

Lipoprotein lipase (LPL) is a multifunctional protein that catalyzes the hydrolysis of plasma triglycerides, releasing free fatty acids, which play critical roles in the metabolism and transport of lipids. The transcription of *LPL* in response to cell types and regulatory factors is a complex process that starts with its promoter. In previous studies, several proximal regulatory elements within the human *LPL* promoter were individually characterized. This study was designed to characterize the effect of 12 proximal regulatory elements as a combined unit on the transcriptional activity of the *LPL* promoter. The hypothesis was that these proximal regulatory elements collectively result in the optimal transcriptional activity of the human *LPL* promoter. Full and partial *LPL* promoter sequences, which contained and excluded the 12 regulatory elements, respectively, were cloned and inserted into a promoterless luciferase reporter vector. The functional activities of these constructs were tested in vitro using a dual-luciferase reporter assay. Our results showed that HEK-293 cells transfected with the full *LPL* promoter exhibited significantly greater luciferase activity than cells transfected with partial *LPL* promoters. Our results indicate that the proximal regulatory elements within the *LPL* promoter, including four TATA boxes, two Oct-1 sites, one CT element, two C/EBPα sites, one SP1 site, and two cis-acting regions (LP-α and LP-β), are essential for its transcriptional activity.

## 1. Introduction

In eukaryotes, genes transcribed by RNA polymerase II usually contain two distinct types of cis-acting transcriptional regulatory DNA elements. The first is a promoter composed of a core promoter and proximal regulatory elements, typically spanning less than 1 kb. The second type is distal regulatory elements, including enhancers, silencers, and insulators, which can be located up to 1 Mb from the promoter. These cis-acting transcriptional regulatory elements contain recognition sites for trans-acting DNA-binding transcription factors, which can either enhance or repress transcription [1,2,3].

There are multiple methods for assessing regulatory elements within promoter regions that control the transcriptional activities of genes. In silico computational bioinformatics analysis has been widely used for the initial prediction of regulatory elements in promoter regions [4,5,6,7]. After bioinformatics analyses, putative transcriptional activation promoter regions can be further tested by site-directed mutagenesis with luciferase assays [8,9]. Deletion mapping can also be used with reporter assays to characterize promoter region regulatory elements and to identify potential transcription-related binding sites [10]. Alternatively, CRISPR-Cas9-based in vivo genome-editing of regulatory elements can be used with reporter assays to measure promoter activity [11]. Luciferase assays rely on assessing the enzymatic activities of reporter proteins, which reflect the transcriptional activities of their genes in intact cells [12,13]. These assays have been commonly used to analyze the regulation of transcriptional activities by promoters and to examine their regulatory elements [12,14,15,16].

Lipoprotein lipase (*LPL*) is an essential member of the lipase family whose primary role is hydrolyzing ester bonds of water-insoluble molecules in lipid metabolism. LPL dysfunction can lead to hypertriglyceridemia and, subsequently, the development of atherosclerosis [17,18,19,20,21]. On the other hand, LPL overexpression is associated with increased LPL activity, decreased plasma triglycerides, and reduced atherosclerosis [20,22].

The sequence of the *LPL* gene has been characterized in various studies using sequences from humans and many other animal species [23,24,25,26,27,28]. The activity and mRNA expression of *LPL* have been documented in a wide range of tissues, including heart, lung, kidney, skeletal muscle, mammary gland, and adipose tissue. A recent study on the longissimus dorsi muscle of Hu sheep has found that *LPL* is expressed at the highest level in adipose tissue [29]. The mRNA expression of *LPL* in the various tissues has been shown to depend on different factors, such as physiological state, feeding/fasting, cold/heat adaptation, nutrition, and metabolic and transport activities [30]. *LPL* regulation is mechanistically complex and occurs at transcriptional, translational, and posttranslational levels, with differences related to cell type and responses to both regulatory factors and extrinsic factors [19,29,31].

The *LPL* gene is approximately 30 kilobase pairs (kb) in length, is located on chromosome 8p22, and encodes a 475-amino acid enzyme. It consists of ten exons and nine introns. Exon 1 encodes the 5′-untranslated region, the signal peptide, and the first two amino acids of the mature protein, whereas exons 2–9 encode the remaining 446 amino acids, and exon 10 encodes the entire 3′-untranslated region [18,31,32,33]. Several regulatory elements within the promoter of *LPL* have been reported [27,34,35].

Polymorphisms and variants within the human *LPL* promoter have been found to be associated with several diseases. For example, a T->C mutation at nucleotide (nt) −39, part of the binding site of the transcription factor Oct-1, lowered *LPL* promoter activity and was associated with hyperlipidemia [36]. The −93T/G mutation was associated with decreased plasma triglyceride levels and increased promoter activity in a human adrenal cell line [10]. The −93 T< G promoter gene polymorphism of *LPL,* a rare variant, has been found to be associated with obesity, while the rare variant −53 G<C is associated with insulin resistance [37]. Other studies have shown that *LPL* promoter regions exhibit different methylation states associated with different blood lipid profiles [38]. Inactivation of the *LPL* promoter by CpG island methylation has been linked to human prostate cancer [39]. A recent review in 2024 reported that genetic studies have identified over 100 loss-of-function and functionally heterogeneous mutations in *LPL* associated with the development of *LPL* deficiency in individuals with Familial Chylomicronemia Syndrome [21].

Deletion mapping, mutagenesis, and reporter assays have been used to analyze the regulatory elements within the *LPL* promoter. For example, different promoter deletion mutants were cloned and inserted into reporter gene constructs and transiently transfected into preadipocytes, and the results showed that LP-α and LP-β are necessary for the differentiation-linked induction of the *LPL* gene during adipogenesis [35]. Mutagenesis testing with a mobility shift assay revealed that two regulatory elements, specificity proteins 1 and 3 (Sp1 and Sp3), transactivate the human lipoprotein lipase gene promoter by binding to a conserved 5′-CCTCCCCCC-3′ motif, which is referred to as the CT element [40]. A recent study involving deletion of the human *LPL* promoter with a luciferase assay revealed a proximal region with a binding site for forkhead box transcription that is important for hepatic cell activity: in vitro silencing of forkhead box transcription reduced the mRNA and protein levels of LPL [22].

While some previous studies focused on the characterization of an individual regulatory element and its effect on the activity of the human *LPL* promoter, this study focused on the characterization of the 12 proximal regulatory elements as a unit, with the assumption that these proximal regulatory elements collectively induce the optimal transcriptional activity of the human *LPL* promoter. This objective was achieved by cloning and inserting the full promoter region, which included the 12 regulatory elements, and a partial promoter region, which excluded any regulatory elements, into a promoterless luciferase vector. The transcriptional activities of the recombinant vectors were then tested in vitro using a human embryonic kidney (HEK) cell line.

## 2. Materials and Methods

### 2.1. Sample Description and Consent to Participate

Ten DNA samples from healthy controls were used to clone the target regions at the *LPL* promoter. At healthcare centers in Kuwait, certified nurses collected blood samples in EDTA tubes from eligible individuals. These individuals were required to be Kuwaiti nationals over 18-years-old and willing to provide blood for a fasting lipid profile. The study excluded individuals diagnosed with metabolic disorders, Type 2 diabetes mellitus, or heart disease, as well as those taking medication that could impact plasma lipid levels and those who declined to give informed consent. Sample details have been previously documented [33]. The sample and medical data collection protocol and informed consent forms used were in accordance with the revised version (2000) of the 1975 Helsinki guidelines and were obtained with ethical approval from the Ministry of Health, Kuwait.

**Figure 1:**
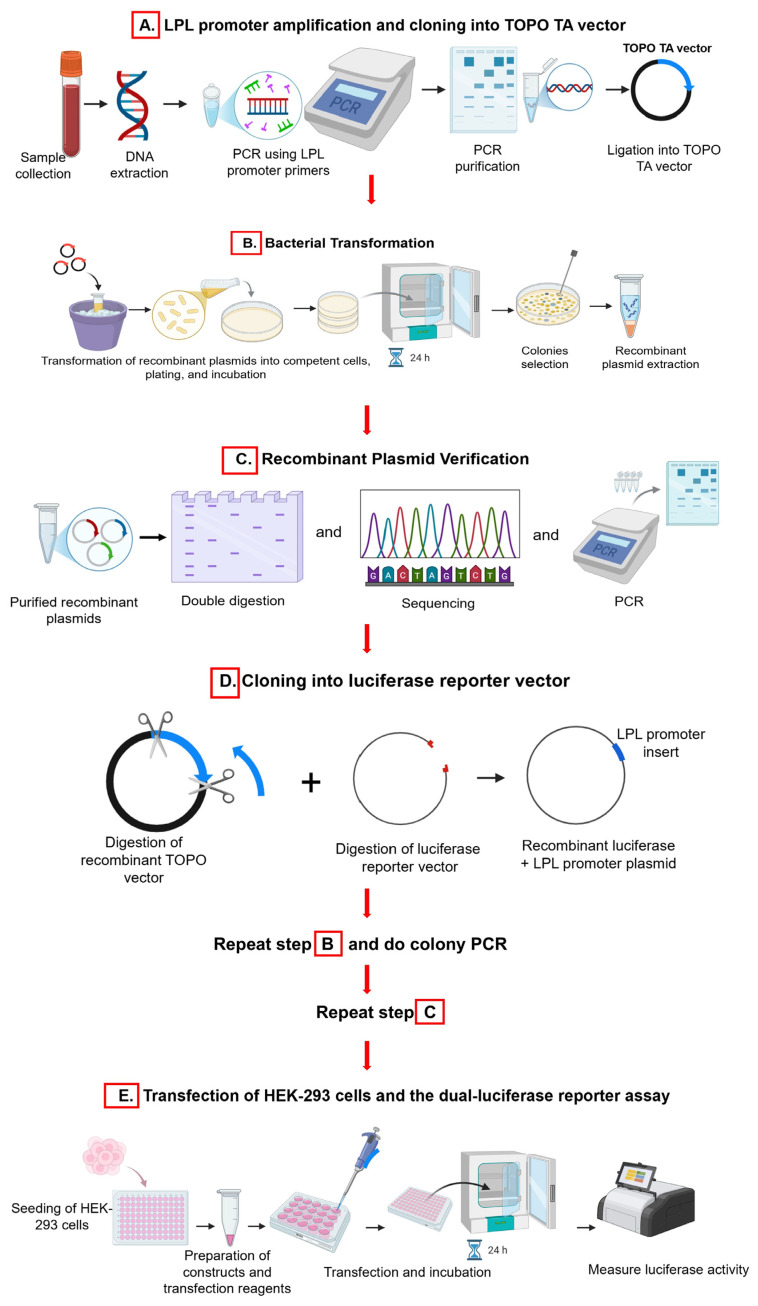
Overview: A schematic representation of the methods used for this study (created with Biorender.com) on 14 October 2024. Refer to Supplementary Figure S1 for the colony PCR.

The methods used in this study for the upcoming Section 2.2, Section 2.3, Section 2.4 and Section 2.5 are illustrated in Figure 1.

### 2.2. Primer Design for Amplifying Full and Partial Promoter Sequences

**Figure 2:**
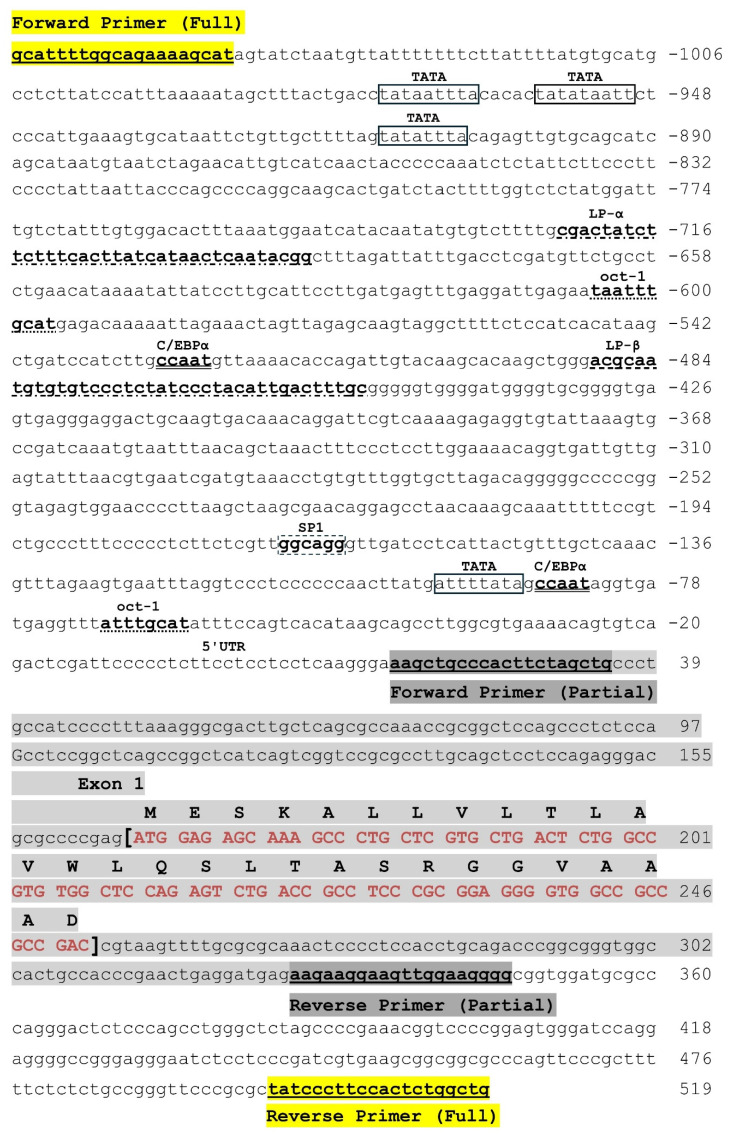
The 12 key regulatory motifs in the 5′ promoter region of the *Homo sapiens LPL* gene. The numbering is relative to the first nucleotide of the first exon (+1). The primers used to amplify the full promoter sequence are underlined and highlighted in yellow and the primers used to amplify the partial promoter sequence are underlined and highlighted in dark gray. The capital letters represent the amino acids encoded by exon 1 (highlighted in red) (Sequence was adapted from the human genome assembly reference sequence NG_008855.2).

Primers were designed to amplify partial (332 bp) and full (1300 bp) promoter regions (Figure 2, Supplementary Tables S1 and S2). The primers used to amplify the full promoter sequence spanned the full sequence illustrated in Figure 2, starting with the forward primer from −1044/−1063 and ending with the reverse primer from +500/+519. The primers used to amplify the partial promoter sequence spanned the small region, starting with the forward primer from +16/+35 and ending with the reverse primer from +328/+347. Primers have been generated using Primer3 and validated with the Primer-BLAST program.

### 2.3. Subcloning and Insertion into the pCR 2.1-TOPO TA Vector

Genomic PCR amplifications were carried out using Platinum™ Green Hot Start PCR 2X Master Mix (13001012, Invitrogen^TM^, Thermo Fisher Scientific, Waltham, MA, USA). The PCR conditions were as follows: initial denaturation at 94 °C for 2 min; 35 cycles of denaturation at 94 °C for 30 s, annealing at 55 °C for 30 s, and extension at 72 °C for 90 s (full promoter)/30 s (partial promoter); and a final extension step at 72 °C for 10 min. The PCR products were purified using a PureLink^®^ Quick Gel Extraction and PCR Purification Combo Kit (catalog no. K220001; Invitrogen ^TM^, Thermo Fisher Scientific, Waltham, MA, USA) and cloned immediately into the pCR 2.1-TOPO TA vector (K450040; Invitrogen, Carlsbad, CA, USA) following the manufacturer’s protocol. Bacterial transformation was carried out using the heat-shock method according to the manufacturer’s instructions. Plasmid purification was performed using a GeneJET plasmid miniprep kit (K0502, Thermo Scientific^TM^, Thermo Fisher Scientific) according to the manufacturer’s protocol. The size and orientation of the inserted fragments in each plasmid were confirmed first by PCR amplification (Supplementary Table S1), second by restriction endonuclease digestion using the EcoR1-HF restriction enzyme (10,000 units; R3101S, New England Biolabs, Ipswich, MA, USA) as described in the manufacturer’s protocol, and third by Sanger DNA sequencing using the BigDye^TM^ Terminator v.3.1 Cycle Sequencing Kit (4337458, Applied Biosystems^TM^, Waltham, MA, USA) as described in the manufacturer’s protocol.

### 2.4. Cloning and Insertion into the Promoterless Luciferase Reporter Vector Plasmid (pGL4.10[luc2])

Full and partial promoter regions were obtained by digesting the 2.1-TOPO TA vectors using the restriction enzymes Sac-HF (R0156S, New England Biolabs, Ipswich, MA, USA) and XhoI (R0146S, New England Biolabs). The targeted regions were cloned and inserted separately into the predigested pGL4.10 [luc2] vector between the Sac and XhoI restriction sites using T4 DNA ligase (M0202S, New England Biolabs, Inc., Beverly, MA, USA). The recombinant vectors were subsequently transformed into chemically competent *E. coli* TOP10 cells. Colony PCR was employed (Supplementary Figure S1 and Supplementary Table S2) to screen for large numbers of positive colonies to save time. Positive clones were grown overnight, after which plasmids were extracted using a GeneJET plasmid miniprep kit (K0502, Thermo Scientific^TM^) according to the manufacturer’s protocol. The size and orientation of the inserted fragments were confirmed by PCR amplification (Supplementary Table S2) and DNA sequencing.

### 2.5. The Transfection of HEK-293 Cells and the Dual-Luciferase Reporter Assay

Human embryonic kidney 293 (HEK-293) cells (ECACC Cat. No. 85120602) were cultured in filtered Dulbecco’s modified Eagle’s medium (DMEM) (Gibco, Grand Island, NY, USA) supplemented with 10% fetal bovine serum (FBS) (10082139, Gibco, Grand Island, NY, USA) and 1% penicillin-streptomycin (15140148, Gibco) at 37 °C and 5% CO_2_. HEK-293 cells were seeded at a density of 1 × 10^4^ cells/well in 96-well plates (92096, TPP Techno Plastic Products AG, Zollstrasse 7, Zürich, Switzerland) and grown overnight in Opti-MEM reduced serum medium (31985062, Gibco, Grand Island, NY, USA) under optimal growth conditions. At 90% confluence, the cells were cotransfected with 100 ng of each recombinant pGL4.10 [luc2] vector and 20 ng of the control Renilla vector (E2231; Promega, Madison, WI, USA) using Lipofectamine^TM^ Transfection Reagent (18324012; Invitrogen, Eugene, OR, USA) according to the manufacturer’s instructions. A dual-luciferase reporter assay system (E1910, Promega, Madison, WI, USA) was used according to the manufacturer’s protocol, and an automated microplate reader (430-1280, Clariostar, Ortenberg, Germany) was used for this experiment. Data analysis has been documented in the literature [41] and was followed in this study. The relative firefly/Renilla luciferase values were calculated for each sample, and the mean ratios of biological replicates were obtained. The experimental sample’s mean value was divided by the vector control mean to determine the fold enrichment of firefly/Renilla luciferase activity. Each experiment included three biological replicates and at least three data sets. The experiments were repeated three times under similar conditions to ensure consistency and reproducibility. The mean fold activation was calculated from multiple experiments for each experimental plasmid [41]. Statistical analyses were performed using GraphPad Prism 9 (GraphPad Software, Inc., San Diego, CA, USA). Values of *p* ≤ 0.05 (*) and *p* ≤ 0.01 (**) values were considered to indicate statistical significance. Values were compared between samples using two-way ANOVA, followed by Tukey’s multiple comparison test.

## 3. Results

### 3.1. The Proximal Regulatory Elements of the Human LPL Promoter

Figure 2 shows the 12 key regulatory elements within the promoter region of the human *LPL* gene. This was accomplished by aligning the human genome assembly sequence (reference NG_008855.2) with previously reported sequences [34,35]. In silico analysis was conducted using the online open-access databases JASPAR and LASAGNA-search to confirm the regulatory elements [42,43]. DNA samples from all participating patients were obtained through Sanger sequencing and compared to the sequence shown in Figure 2.

The analysis revealed the following consensus sequences for the transcription factors: four TATA boxes (−90/−97; −907/−914; −964/−972; −950/−958), two octamer-binding protein-1 (Oct-1) sites (−62/−69; −600/−605), one evolutionarily conserved CCTCCCCCC motif known as the CT element (−106/−114), two CCAAT enhancer-binding protein alpha (C/EBPα) sites (−84/−88; −524/−528), and one SP1 site (−63/−69). Moreover, two cis-acting regions known as LP-α and LP-β were found: LP-α (CGACTATCTTCTTTCACTTATCATAACTCAATACGG), located at −689/−724, and LP-β (ACGCAATGTGTGTCCCTCTATCCCTACATTGACTTTGC), located at −452/−489 (Figure 2).

### 3.2. The Construction of Recombinant Luciferase Vectors for Testing the Activity of the LPL Promoter

Full and partial protomer sequences were successfully subcloned and inserted into transient pCR 2.1-TOPO TA vectors (Supplementary Figure S2a–c). PCR amplification was performed using primers predesigned from the TOPO vector (Supplementary Table S1) to confirm the integrity of the plasmids (Supplementary Figure S2d,e).

Full and partial promoters were digested from the TOPO TA vectors and cloned and inserted into the promoterless luciferase reporter vector (pGL4.10[luc2]). Ligation was performed at 1:3 and 1:5 vector-to-insert ratios, and the transformation efficiencies were compared (Supplementary Figure S3). A 1:3 ratio generated fewer but larger colonies than a 1:5 ratio, which generated more but smaller colonies.

Colony PCR was performed by combining partial bacterial cells from 8–10 positive clones (Supplementary Figure S1). A positive clone mixture was selected from the pool based on PCR amplification (Supplementary Figure S4) and subjected to plasmid extraction for further analysis.

**Figure 3:**
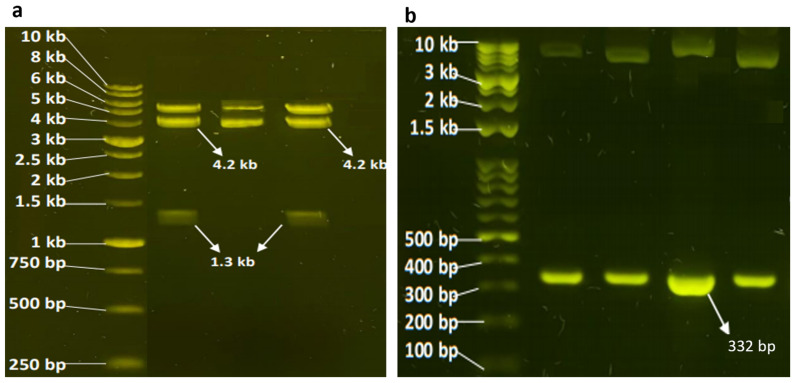
Agarose gel showing the resolved plasmids harboring full (**a**) or partial (**b**) *LPL* promoters cloned and inserted into the pGL4.10[luc2] luciferase vector. The plasmids were digested with SacI and XhoI. The fragments were resolved on a 1% agarose gel prestained with 10 mg/mL SYBR Safe and imaged under UV light.

**Figure 4:**
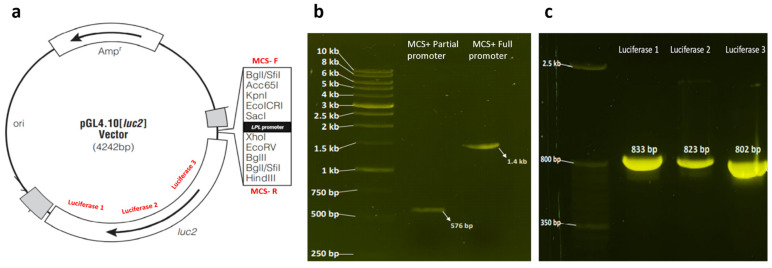
(**a**) A plasmid map of the promoterless pGL4.10[luc2] luciferase vector showing the regions amplified from the vector (modified from Promega; the red labels on the map indicate the primer regions). Agarose gel showing the full and partial promoter inserts amplified from the recombinant luciferase vectors using MCS primers in (**b**) and luciferase-specific primers in (**c**).

Recombinant vectors harboring the full or partial promoter sequence + luciferase reporter vector (pGL4.10[luc2] were tested, first, by double digestion with SacI and XhoI, which revealed a perfect match of the sizes of the inserted cloned sequences (Figure 3a,b); second, by amplification with PCR using primers predesigned around the vector (Supplementary Table S2 and Figure 4a–c); and third, by sequencing (Supplementary Figure S5a,b).

### 3.3. HEK-293 Cells Transfected with the Full Promoter Sequence Exhibit Greater Luciferase Activity than Cells Transfected with the Partial Promoter Sequence

**Figure 5:**
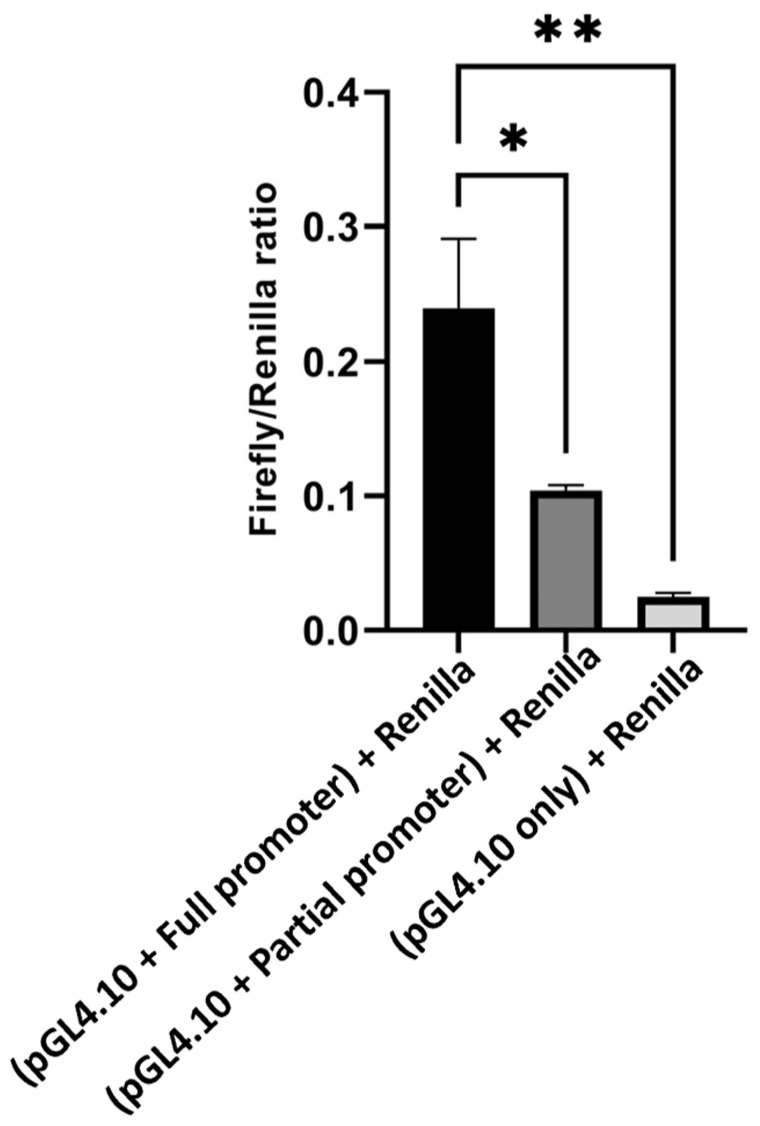
A comparison of luciferase activity obtained using the different tested vectors to determine the effect of the full and partial *LPL* promoters on pGL4.10 gene expression in HEK-293 cells. This effect was measured as the mean of the firefly/*Renilla* luciferase ratio. The results are presented as the means from three independent experiments conducted in triplicate (* *p* ≤ 0.05, ** *p* ≤ 0.01).

To test the effect of the proximal regulatory elements of the *LPL* promoter on activity, recombinant luciferase vectors carrying full or partial promoter sequences were transfected into HEK-293 cell lines along with the Renilla vector as an internal control. The relative luciferase activity was determined using a dual-luciferase reporter gene. Strong induction of luciferase activity was observed for cells transfected with the pGL4.10[luc2] luciferase vector harboring the full promoter sequence compared to cells transfected with a partial promoter sequence, which resulted in a significant decrease in luciferase activity (* *p* ≤ 0.05) (Figure 5). Cells transfected with the pGL4.10[luc2] luciferase promoterless vector and Renilla luciferase exhibited a sudden decrease in luciferase activity (** *p* ≤ 0.01). These results demonstrated that the proximal regulatory elements within *LPL* promoters are essential for modulating the promoter activity of *LPL.*

## 4. Discussion

In this study, the protocol developed and established was demonstrated as a reliable system for assessing the transcriptional activity of the human *LPL* promoter in the presence of the non-mutated sites of the 12 regulatory elements modified with a luciferase assay and for the assessment of the transcriptional activity of the human *LPL* promoter in the presence of the 12 non-mutated regulatory elements. Previous reports on *LPL* have also used luciferase assays for association studies. For example, the luciferase gene was used as a reporter to test the promoter activity of the −93T/G variants. The results showed that the −93T/G mutation in the *LPL* promoter was associated with decreased plasma triglyceride levels, and the luciferase assay confirmed the increase in *LPL* promoter activity in vitro when the wild-type allele was present [10]. Luciferase assays and deletion analysis of the human *LPL* promoter also revealed a binding site for the forkhead box transcription factor, which is essential for *LPL* promoter activity in hepatic cell lines [22].

In our study, the full promoter sequence covered a region from −1006/+518, which included the 12 proximal regulatory elements, while the partial promoter sequence covered a region from +39/+360 without any regulatory elements. The regulatory elements within the full promoter region included four TATA boxes, two Oct-1 sites, one CT element, two CCAAT enhancer-binding protein alpha (C/EBPα) sites, one SP1 site, and two cis-acting regions known as LP-α and LP-β. Some of these regulatory elements within the human *LPL* promoter have been characterized individually with regard to their effect on human *LPL* activity [22,35,40].

These previous studies used serial deletions of the human *LPL* promoter region to characterize the effects of individual regulatory elements on the transcriptional activity of the *LPL* promoter. For example, deletion analysis of the human *LPL* promoter revealed that the proximal region of the *LPL* promoter contains the regulatory element Oct-1, which is an important binding site for the forkhead box transcription factor FOXA2/HNF-3β and is essential for hepatic cell activity [22]. The two cis-regulatory elements LP-α and LP-β were shown to be important for binding hepatic nuclear factor (NF) 3-like proteins, which is essential for the gradual activation of the *LPL* gene during adipocyte development in vitro [35]. The deletion or mutation of the CT element required for the binding of transcription factors SP1 and SP3 in the human lipoprotein *LPL* promoter led to a reduction of about 70–80% in promoter activity in a human monocytic leukemia cell line [40].

These previous studies have been both outdated and limited and have focused on individual regulatory elements and their effects on the activity of the human *LPL* promoter. In contrast, our recent study examines the combined effect of the 12 proximal regulatory elements and how they may affect the activity of the human *LPL* promoter. Therefore, full (−1006/+518) and partial (+39/+360) *LPL* promoter sequences were cloned and inserted into a luciferase reporter vector lacking eukaryotic promoter elements, and their ability to drive luciferase expression after transfection into HEK-293 cell lines was assessed. Our sequence alignment of the cloned full and partial *LPL* promoter sequences revealed no mutations in any of the bases of the cloned regions. Additionally, there were no differences in the DNA sequences among the blood samples collected for this study. Our results showed that HEK-293 cells transfected with the full *LPL* promoter, which includes 12 different proximal regulatory motifs, exhibited significantly greater luciferase activity than cells transfected with the partial *LPL* promoter, which contains no regulatory motifs. Moreover, the results of the transcriptional activities were also consistent across the samples. These results indicate that the 12 proximal regulatory elements within the full *LPL* promoter contribute significantly to the transcriptional activity of *LPL* promoter.

The HEK-293 cell line has been chosen for this study for several reasons. It is known for its rapid growth and ease of cultivation. Additionally, it is easy to passage and has a high success rate for transfection. The HEK-293 cell line is widely used among human cell lines due to its high transfectivity, quick growth, and ability to thrive in a serum-free suspension culture [44,45].

A luciferase assay has also been routinely used to study the activity of cloned promoter DNA fragments in vitro in eukaryotic cell lines, providing rapid and sensitive quantification of the cloned DNA fragments [12,41,46,47]. For example, in the human insulin growth factor promoter, recombinant luciferase plasmids containing the 5′ UTR of exon 1 and a 1630 nt segment of the 5′ flanking sequence stimulated luciferase activity nearly 70 times greater than that in cells transfected with a promoterless control plasmid [48]. In another example, the luciferase assay was used to identify two regions that promote *REV7* expression in the upstream promoter region of the REV7 gene [8]. Similarly, a luciferase assay was used to analyze the promoter activity of SCC antigen, which is a tumor marker for squamous cell carcinoma [49]. Recently, a similar study also utilized a luciferase assay to examine the promoter specific to porcine adipose tissue [50]. These reported findings are consistent with our results.

Globally, the rates of obesity and diabetes have risen, and heart disease remains the primary cause of death associated with these conditions [51]. High-fat diets leading to insulin resistance in obesity is a significant risk factor for diabetes and cardiovascular disease. Several studies have shown that increased LPL activity can ameliorate obesity; overexpression of LPL protects against diet-induced hypertriglyceridemia and hypercholesterolemia [52,53]. For example, increased LPL activity in the skeletal muscle of rats reduced obesity and insulin resistance in obese rats [52]. Another study has shown that a modest increase in LPL expression in adipose tissue was linked to improved glucose and insulin tolerance, positively impacting total body-energy metabolism [54]. A recent review emphasized the importance of the LPL-mediated breakdown of lipoproteins as a key source of fatty acids for energy production in the heart. It also examined the various pharmaceutical strategies that have been investigated to lower cardiovascular risk by targeting LPL [51]. All these studies support our objectives in developing a protocol that allows the analysis of the full promoter versus a partial promoter in the optimal transcriptional level of *LPL*. The outcome of our study has provided the means to investigate the role of the full promoter and partial promoters in various tissues to identify the regulatory mechanisms of *LPL* expression.

In addition, a previous study conducted by one of our research team members has reported that prolonged elevation of plasma lipid levels can lead to dyslipidemia, which may result in further complications such as coronary heart disease (CHD) or atherosclerosis. The study has shown that variants in the LPL gene locus have been found to contribute to fluctuations in plasma lipid levels, particularly triglycerides (TG) and high-density lipoprotein cholesterol (HDL-C), and these genetic variants may directly affect gene expression or interact with other variants to exert their influence [33]. Therefore, understanding the transcriptional activity of LPL is essential as it can help in the comprehension of widespread diseases, potentially leading to the delay, prevention, or treatment of human diseases.

## 5. Conclusions

In conclusion, few studies have focused on the regulatory elements within the human *LPL* promoter. In previous studies, several proximal regulatory elements within the human *LPL* promoter were characterized individually. This study aimed to characterize the effect of the 12 proximal regulatory elements on the transcriptional activity of the *LPL* promoter. These regulatory elements have been previously reported to exist within the human *LPL* promoter. However, their collective activity as a unit was not examined prior to this study. We have shown that these 12 proximal regulatory elements are important for the induction of optimal transcriptional activity at the *LPL* promoter.

Some of these 12 regulatory elements might be more essential than others, or the presence of one or more of them might inhibit or enhance the activity of the others. It is also possible that these elements behave differently in different tissues to produce tissue-specific isoforms of the LPL enzyme. Therefore, in future studies, it will be necessary to test the individual functional roles of regulatory elements in different tissues. These tests can be performed by site-directed mutagenesis and deletion analysis of individual regulatory elements, followed by the construction of mutated forms of *LPL* promoters to determine their functional activities with luciferase assays. Additionally, future studies could also examine mutations in the regulatory elements of the LPL promoter and their implications for specific human diseases. In this study, we tested the activity of these compounds in the HEK-293 cell line; other tissue cell lines, such as adipocytes or liver tissue, might produce different results. Therefore, further investigation by cloning and luciferase assays, as described in this study, is warranted.

## Data Availability

The original contributions presented in the study are included in the article or the  Supplementary Materials, and further inquiries can be directed to the corresponding author.

## Supplementary Materials

The following supporting information can be downloaded at https://www.mdpi.com/article/10.3390/cimb46110788/s1, Figure S1: Illustration of the colony formation PCR procedure used to select for positive colonies. Images were created with BioRender; Figure S2: (a) Map of the pCR 2.1-TOPO TA vector (modified from Thermo Fisher Scientific, Invitrogen). (b,c) The DNA fragments (full and partial *LPL* promoters) were cloned and inserted into the pCR 2.1-TOPO TA pGL4.10 vector. (d,e) Regions around the pCR 2.1-TOPO TA pGL4.10 recombinant vector were amplified with various primers (the red label on the map indicates the primer region); Figure S3: Transformation efficiency of the recombinant luciferase vector on agar plus ampicillin. (a) Nontransformed cells. (b,c) Transformed cells at a 1:3 vector-to-insert ratio (b) and 1:5 vector-to-insert ratio (c). Figure S4: Agarose gel showing the colony-PCR results for selecting positive clones containing plasmids with a full promoter+ luciferase vector in (a) and plasmids with a partial promoter+ luciferase vector in (b); Figure S5: An example of the electrophoretograms of the desired *LPL* promoters cloned and inserted into the pGL4.10[luc2] luciferase vector using the forward primers from the luciferase vector region. The blue bars in (a,b) indicate that the quality of the generated nucleotide sequence was above 95%.; Table S1: The sequences used for cloning into the transient pCR 2.1-TOPO TA vector; Table S2: The sequences used for cloning into the promoterless luciferase reporter vector plasmid (pGL4.10[luc2]).

## Abbreviations

C/EBPα, CCAAT enhancer-binding protein alpha; DMEM, Dulbecco’s modified Eagle’s medium; FBS, Fetal bovine serum; HEK, Human embryonic kidney; LPL, Lipoprotein lipase; MCS, Multiple cloning Site; NF, Nuclear factor; Oct-1, Octamer-binding protein-1.
