# Supplementary material for: A Set of Proximal Regulatory Elements Contribute to the Transcriptional Activity of the Human Lipoprotein Lipase Promoter
Source: Curr Issues Mol Biol. 2024 Nov 18;46(11):13209–22. doi: 10.3390/cimb46110788 (PMC11592473; doi:10.3390/cimb46110788)
Supplement: Supplementary file 1 [file cimb-46-00788-s001.zip › cimb-3321494-supplementary.pdf]

Supplementary Materials

Supplementary Table S1. The sequences used for cloning into the transient pCR 2.1-TOPO TA vector

Name of the sequence used	Forward and reverse primers (5′–3′)	Expected product size
Partial *LPL* Promoter	F: AAGCTGCCCACTTCTAGCTG<br>R: CCCCTTCCAACTTCCTTCTT	332 bp
Full *LPL* Promoter	F: GCATTTTGGCAGAAAAGCAT<br>R: CAGCCAGAGTGGAAGGGATA	1300 bp
M13 Forward and Reverse	F: GTAAAACGACGGCCAG<br>R: CAGGAAACAGCTATGAC	Depends on the insert size
Topo 1 region of pCR 2.1-TOPO TA	F: ATACGCAAACCGCCTCTCC<br>R: CCGAAATCGGCAAAATCCCT	1150 bp
Topo 2 region of pCR 2.1-TOPO TA	F: CATCGCCCTGATAGACGGTT<br>R: ATGCGATGTTTCGCTTGGTG	925 bp
Topo 3 region of pCR 2.1-TOPO TA	F: TGCTCGACGTTGTCACTGAA<br>R: AGTAAGTTGGCCGCAGTGTT	945 bp
Topo 4 region of pCR 2.1-TOPO TA	F: CGGTCGCCGCATACACTATT<br>R:TGCTACAGAGTTCTTGAAGTGT	961 bp
Topo 5 region of pCR 2.1-TOPO TA	F: GCGTCAGACCCCGTAGAAAA<br>R: TCCGCTCACAATTCCACACA	980 bp

Supplementary Table S2. The sequences used for cloning into the promoterless luciferase reporter vector plasmid (pGL4.10[luc2])

Name of the sequence used	Forward and reverse primers (5′–3′)	Expected product size
Partial *LPL* Promoter	F: AAGCTGCCCACTTCTAGCTG<br>R: CCCCTTCCAACTTCCTTCTT	332 bp
Full *LPL* Promoter	F: GCATTTTGGCAGAAAAGCAT<br>R: CAGCCAGAGTGGAAGGGATA	1300 bp
MCS of (pGL4.10[luc2])	F: CTAACTGGCCGGTACCTGAG<br>R: ATGATCTGGTTGCCGAAGAT	Depends on the insert size
Luciferase 1 of (pGL4.10[luc2])	F: ACAAAACCATCGCCCTGAT<br>R: GTCGAAGATGTTGGGGTGTT	833 bp
Luciferase 2 of (pGL4.10[luc2])	F:GGCTGAAGAGCCTGATCAAA<br>R:CGCCTTTGAGTGAGCTGATA	823 bp
Luciferase 3 of (pGL4.10[luc2])	F: CTGCCTATTTCGTTCGTCCA<br>R:TGTCGCACTCATTCCCTTCT	802 bp

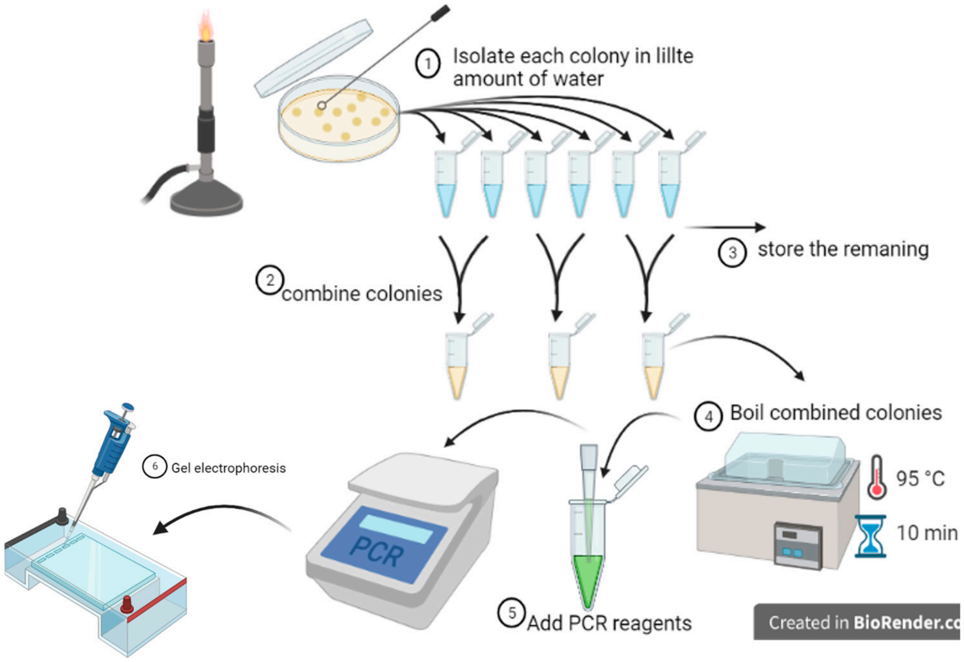

Supplementary Figure S1. Illustration of the colony formation PCR procedure used to select for positive colonies. Images were created with BioRender

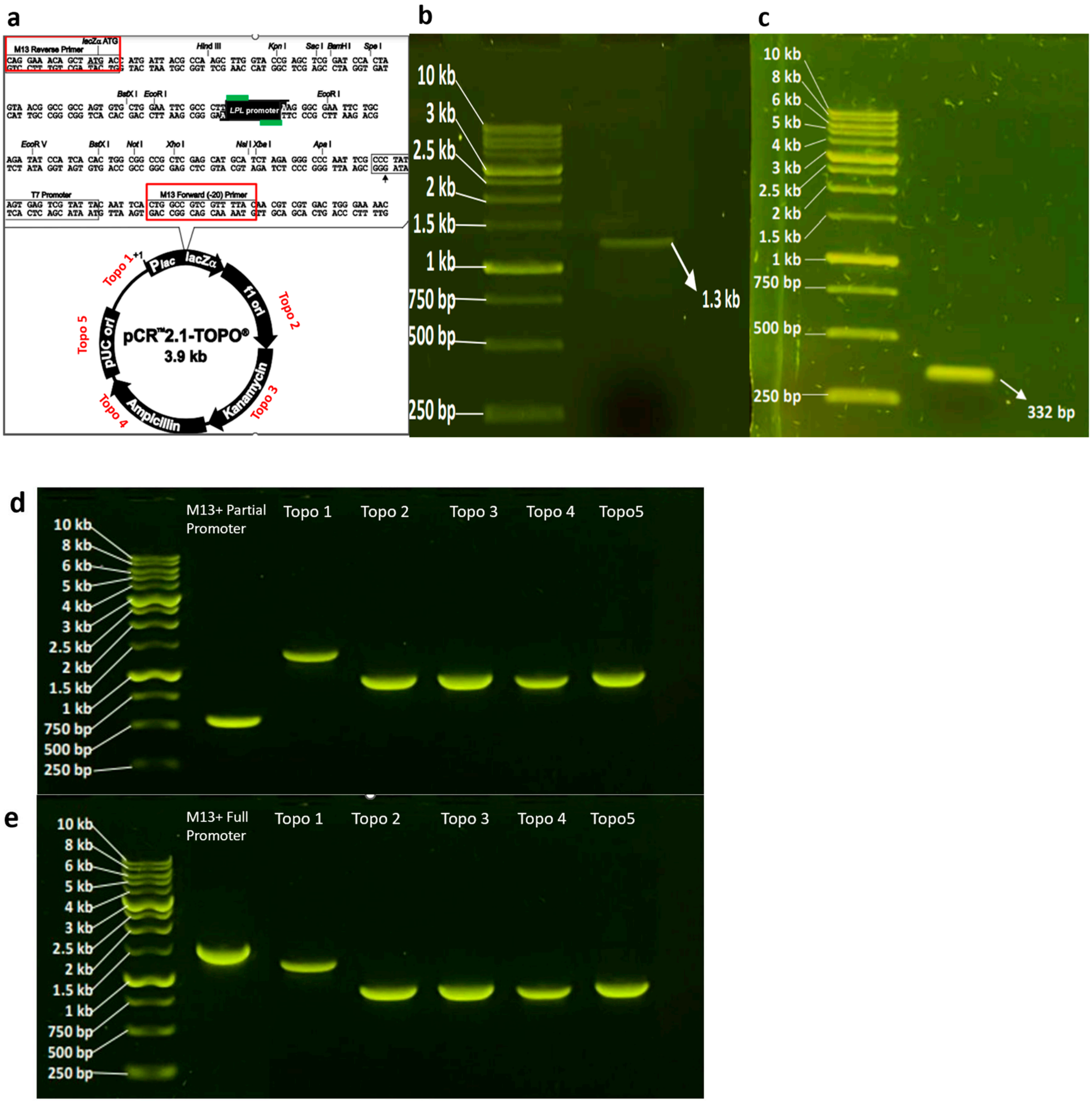

Supplementary Figure S2. (a) Map of the pCR 2.1-TOPO TA vector (modified from Thermo Fisher Scientific, Invitrogen). (b) and (c) The DNA fragments (full and partial *LPL* promoters) were cloned and inserted into the pCR 2.1-TOPO TA pGL4.10 vector. (d) and (e) Regions around the pCR 2.1-TOPO TA pGL4.10 recombinant vector were amplified with various primers (the red label on the map indicates the primer region).

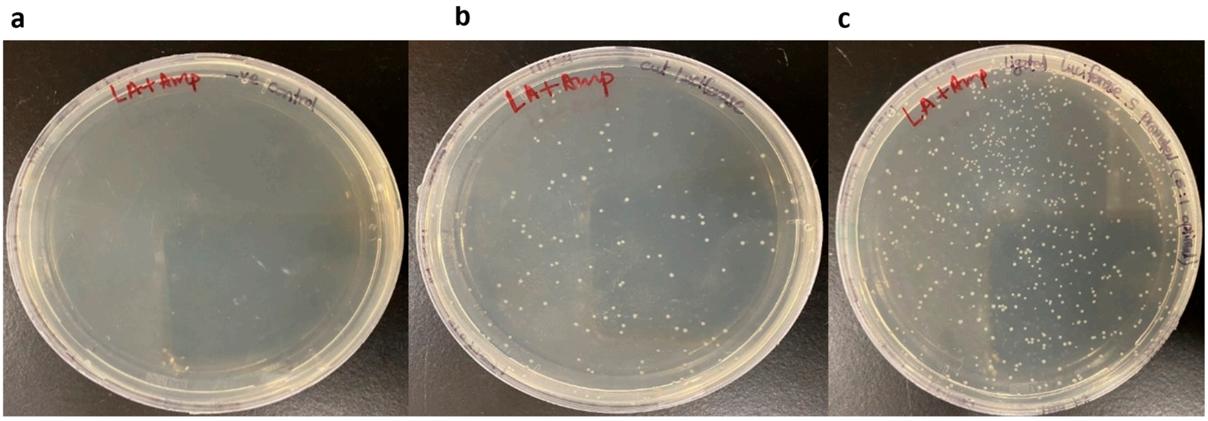

Supplementary Figure S3. Transformation efficiency of the recombinant luciferase vector on agar plus ampicillin. (a) Nontransformed cells. (b) and (c) Transformed cells at a 1:3 vector-to-insert ratio (b) and 1:5 vector-to-insert ratio (c).

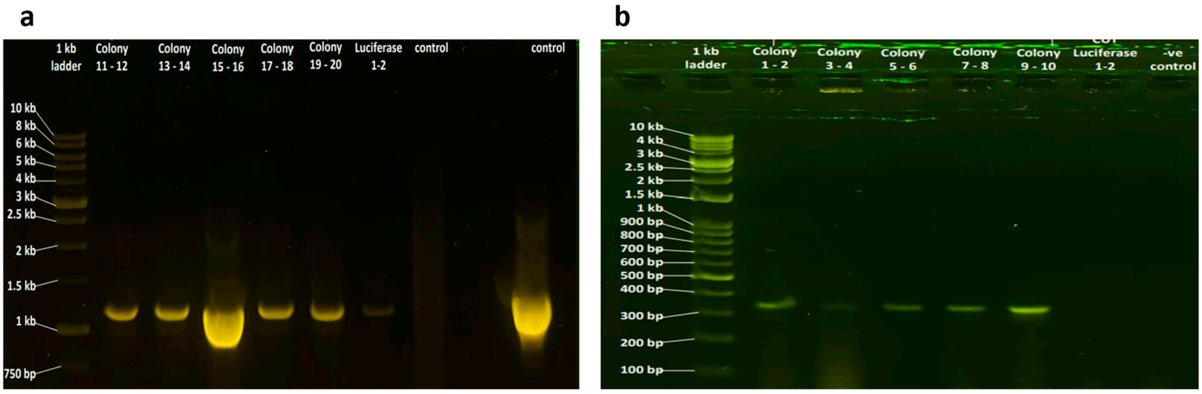

Supplementary Figure S4. Agarose gel showing the colony-PCR results for selecting positive clones containing plasmids with a full promoter+ luciferase vector in (a) and plasmids with a partial promoter+ luciferase vector in (b).

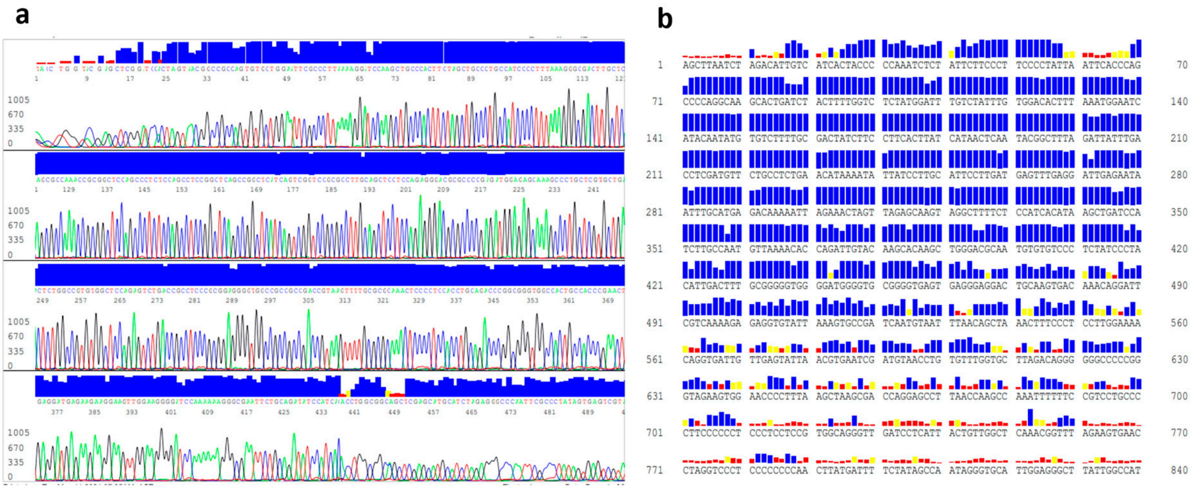

Supplementary Figure S5. An example of the electrophoretograms of the desired *LPL* promoters cloned and inserted into the pGL4.10[luc2] luciferase vector using the forward primers from the luciferase vector region. The blue bars in (a) and (b) indicate that the quality of the generated nucleotide sequence was above 95%.
